# Gene Expression Profiling of H9c2 Myoblast Differentiation towards a Cardiac-Like Phenotype

**DOI:** 10.1371/journal.pone.0129303

**Published:** 2015-06-29

**Authors:** Ana F. Branco, Susana P. Pereira, Susana Gonzalez, Oleg Gusev, Albert A. Rizvanov, Paulo J. Oliveira

**Affiliations:** 1 CNC—Center for Neuroscience and Cell Biology, UC-Biotech Building, Biocant Park, University of Coimbra, Cantanhede, Portugal; 2 Department of Life Sciences, Largo Marques de Pombal, University of Coimbra, Coimbra, Portugal; 3 Stem Cell Aging Group, Spanish National Cardiovascular Research Center (CNIC), Madrid, Spain; 4 Institute of Fundamental Medicine and Biology, Kazan Federal University, Kazan, Russia; Childrens Hospital Los Angeles, UNITED STATES

## Abstract

H9c2 myoblasts are a cell model used as an alternative for cardiomyocytes. H9c2 cells have the ability to differentiate towards a cardiac phenotype when the media serum is reduced in the presence of all-trans-retinoic acid (RA), creating multinucleated cells with low proliferative capacity. In the present study, we performed for the first time a transcriptional analysis of the H9c2 cell line in two differentiation states, i.e. embryonic cells and differentiated cardiac-like cells. The results show that RA-induced H9c2 differentiation increased the expression of genes encoding for cardiac sarcomeric proteins such as troponin T, or calcium transporters and associated machinery, including SERCA2, ryanodine receptor and phospholamban as well as genes associated with mitochondrial energy production including respiratory chain complexes subunits, mitochondrial creatine kinase, carnitine palmitoyltransferase I and uncoupling proteins. Undifferentiated myoblasts showed increased gene expression of pro-survival proteins such as Bcl-2 as well as cell cycle-regulating proteins. The results indicate that the differentiation of H9c2 cells lead to an increase of transcripts and protein levels involved in calcium handling, glycolytic and mitochondrial metabolism, confirming that H9c2 cell differentiation induced by RA towards a more cardiac-like phenotype involves remodeled mitochondrial function. PI3K, PDK1 and p-CREB also appear to be involved on H9c2 differentiation. Furthermore, complex analysis of differently expressed transcripts revealed significant up-regulation of gene expression related to cardiac muscle contraction, dilated cardiomyopathy and other pathways specific for the cardiac tissue. Metabolic and gene expression remodeling impacts cell responses to different stimuli and determine how these cells are used for biochemical assays.

## Introduction

Primary cardiomyocytes are fragile and difficult to maintain in culture for long periods. Furthermore, their isolation requires the sacrifice of laboratory animals, which is a serious concern nowadays. Therefore, there is a large demand for the use of proper *in vitro* cardiac-like cell models which can be used in cell biology, electrophysiology and toxicology research. The H9c2 (2–1) myoblast cell line, isolated from ventricular tissue, is currently used *in vitro* as a mimetic for skeletal and cardiac muscle due its biochemical, morphological and electrical/hormonal signaling properties [1, 2]. The H9c2 cell line was initially isolated from the ventricular part of a BDIX rat heart [2]. Thirteen days after fecundation, cells were isolated and immortalized. By selective serial passages, the different adhesion kinetics of the heterogeneous isolated fraction led to the separation of the different components in the culture dish. In this stage, cells are still not fully differentiated into adult cardiomyocytes but are already predestinated, leading to the appearance of several cardiomyocyte-specific markers. One important feature of this embryonic cell line is its ability to differentiate from mono-nucleated myoblasts to myotubes when cultured in a low serum concentration media, getting an elongated shape and positioning in a parallel fashion [3]. During the differentiation process, cells obtain mostly a skeletal muscle phenotype, as evidenced by cell type-specific differentiation markers such as myogenin and MyoD [4]. Furthermore, Ménard et al. demonstrated that addition of all-trans retinoic acid (RA) to a 1% serum media induces a predominant presence of cells presenting an adult cardiac muscle phenotype, characterized by the overexpression of the alpha-1 subunit of L-type calcium channels [4]. H9c2 cells do not present contractile activity, even when differentiated. However, H9c2 cells and isolated neonatal cardiomyocytes respond similarly to several stimuli including by developing hypertrophic responses [5]. The great majority of studies are performed using undifferentiated H9c2 myoblasts, raising questions on the relevance of the results obtained when compared to primary cardiomyocytes. This is especially pertinent in cardiotoxicity studies, since dose-responses are altered by the cell differentiation state [6, 7]. Because the adult heart tissue contains mostly differentiated cardiomyocytes without proliferative activity, toxicological assessment studies may result in different outcomes depending on the cell differentiation state. The present work extends our previous studies [8–10] by characterizing transcriptome alterations during H9c2 differentiation towards a cardiac-like phenotype. The data was obtained by using an Agilent Rattus norvegicus total genome microarray with some of the significant hits confirmed by Western blotting. The results are very relevant to understand metabolic and signaling alterations occurring during H9c2 cardiomyoblast differentiation, paving the way for a more suitable use of this cell model for different experimental aims.

## Materials and Methods

### Reagents

Bovine serum albumin (BSA), RA, Bradford reagent, DL-Dithiothreitol (DTT), Dulbecco's-modified eagle's medium (DMEM), β-mercaptoethanol 98%, phenylmethylsulfonyl fluoride (PMSF), sulforhodamine B, protease inhibitor cocktail (containing 1mg/ml of leupeptin, antipain, chymostatin and pepstatin A) were obtained from Sigma (Barcelona, Spain). Penicillin, streptomycin, fetal bovine serum (FBS) were purchased from Gibco-Invitrogen (Grand Island, NY). Laemmli buffer, polyvinylidene difluouride (PVDF) membranes and Ponceau solution were obtained from BioRad (Hercules, CA, USA). The ECF detection system was obtained from Healthcare Life Sciences (Buckingamshire, United Kingdom). The fluorescent probes Hoechst 33342, 4',6-diamidino-2-phenylindole (DAPI), tetramethylrhodamine methyl ester (TMRM) were obtained from Invitrogen-Molecular Probes (Eugene, OR, USA). Cell lysis buffer was obtained from Cell Signaling Technology (Leiden, The Netherlands). All reagents used in this work were of the greatest degree of purity commercially available. Ultrapure distilled water was used in the preparation of solutions in order to minimize contamination with metal ions.

#### H9c2 cell culture and differentiation process

The H9c2 cell line obtained from America Tissue Type Collection (Manassas, VA; catalog # CRL-1446) was cultured in DMEM medium supplemented with 1.5 g/L sodium bicarbonate, 10% fetal bovine serum, 100 U/ml penicillin and 100 μg/ml streptomycin in 75 cm^2^ tissue culture flasks at 37°C in a humidified atmosphere of 5% CO_2_. Cells were fed every 2–3 days, and sub-cultured when reaching 70–80% confluence in order to prevent the loss of the differentiation potential. Cell cardiac differentiation was initiated by decreasing the percentage of serum in the media followed by RA supplementation [4]. H9c2 cells were plated at a density of 5,000 cells/ml, for non-differentiated conditions (10% FBS) or at 35,000 cells/ml for cell differentiated conditions (1% FBS + RA) in 75 cm^2^ tissue culture flasks and cultured for one more day in high serum in order to allow for cell attachment. Addition of RA (1 μM) to media containing 1% serum was performed daily in the dark for 5 days. All-trans-RA was prepared in DMSO and stored at -20°C in the dark to avoid degradation.

#### Sulforhodamine B colorimetric assay

The sulforhodamine (SRB) assay was performed to measure cell mass, which is dependent of cell numbers, as previously described [7, 11]. Cells were seeded at a density of 5,000 cells/ml or 35,000 cells/ml in 6 wells plates and allowed to attach for 24 hours. Cells were allowed to proliferate for 6 days in 10% FBS or 1% FBS +RA, after that were fixed with 1% acetic acid in ice-cold methanol during 1 hour at -20°C. Cell number-dependent increase in cell mass was measured by adding 0.5% (w/v) SRB dissolved in 1% acetic acid to each well for 1 hour at 37°C. Unbound dye was washed out with 1% acetic acid. Protein-labeled dye was dissolved in 10 mM Tris-Base solution, pH 10, and the optical density of the resulting solution was measured at 540 nm in a multiplate reader.

#### Western blotting

H9c2 cells were trypsinized, centrifuged for 5 min at 1,000 x g and the pellet washed with cold PBS. Cells were then resuspended in lysis buffer containing sodium orthovanadate, a phosphatase inhibitor, supplemented with 2 mM DTT, 100 μM PMSF and a protease inhibitor cocktail. Extracts were sonicated and stored at -80°C until used. Protein contents were determined by the Bradford method [12] using bovine serum albumin as standard. After denaturation for 5 min at 95°C in Laemmli buffer supplemented with β-mercaptoethanol, equivalent amounts of total protein (25 μg) were separated by electrophoresis in 8%, 12% or 14% SDS-polyacrylamide gels (SDS-PAGE) and electrophoretically transferred to a polyvinylidene difluoride (PVDF) membrane. After blocking with 5% milk or BSA in TBST (50 mM Tris-HCl, pH 8; 154 mM NaCl and 0.1% tween 20) for 2 hours at room temperature or overnight at 4°C, membranes were incubated overnight at 4°C with the specific antibodies: mouse monoclonal anti-TOMM20 (1:1,000), mouse monoclonal anti-creatine kinase (1:1,000), mouse monoclonal anti-lactate dehydrogenase (1:1,000), mouse monoclonal anti-Ndufs4 (1:1,000), rabbit polyclonal anti-Pink1 (1:1,000), mouse monoclonal anti-SERCA2 ATPase (1:500), rabbit polyclonal anti-glutathione peroxidase 1 (1:1,000) and rabbit monoclonal anti-UCP3 (1:1,000), rabbit polyclonal anti-calsequestrin (1:1000); rabbit monoclonal anti-GATA4 (1:1,000); obtained from Abcam (Cambridge, UK); rabbit monoclonal anti-hexokinase II (1:1,000), rabbit polyclonal anti-pyruvate dehydrogenase (1:1,000), rabbit polyclonal anti-p44/42 MAPK (Erk 1/2) (1:1,000); rabbit monoclonal anti-phospho-p44/42 MAPK (Erk 1/2) (1:2000); rabbit monoclonal anti-PI3 kinase class III (1:1,000); rabbit monoclonal anti-CREB (1:1,000); rabbit monoclonal anti-PTEN (1:1,000); rabbit polyclonal anti-PDK1 (1:1000); rabbit polyclonal anti-PI3 Kinase p85 (1:1,000); rabbit monoclonal anti-AKT (pan) (1:1,000) and rabbit monoclonal anti-phospho-AKT (1:1,000) from Cell Signaling (Danvers, MA, USA); rabbit polyclonal anti-phospho CREB (1:1,000) Millipore (Temecula, California, USA) rabbit polyclonal anti-PGC-1α(1:1,000); rabbit polyclonal anti-troponin I (1:500) purchased from Santa Cruz (Dallas, Texas, USA); goat anti-rabbit (1:5,000) and goat anti-mouse (1:5,000) secondary antibodies conjugated with alkaline phosphatase were obtained from Jackson IR Laboratories (Maine, USA). Membranes were incubated with the ECF detection reagent and imaged by using the Versa Doc imaging system (Bio-Rad, Barcelona, Spain). Density of different bands was calculated with Quantity One Software (Bio-Rad). Ponceau staining was used to confirm equal protein loading and to normalize the data, as the differentiation process can affect the amount of housekeeping proteins, such as beta-actin.

#### Vital fluorescence of H9c2 cells by using tetramethylrhodaminemethyl-ester (TMRM) calcein-AM and Hoechst 33342

After the differentiation process, H9c2 cells grown on glass-bottom dishes were incubated with TMRM (100 nM), Hoechst 33342 (1 mg/ml) or calcein-AM (300 nM) at 37°C in the dark. Media was replaced by warm Krebs buffer (1 mM CaCl_2_; 132 mM NaCl; 4 mM KCl; 1.2 mM Na_2_HPO_4_; 1.4 mM MgCl_2_; 6 mM glucose; 10 mM HEPES, pH 7.4) supplemented with 10% FBS. Epifluorescence microscopy images were obtained using a Nikon Eclipse TE2000U microscope and were acquired using Metamorph software (Universal Imaging, Downingtoen, PA).

#### Total RNA extraction

Collection of total RNA extracts was performed following the vendor’s instructions (RNeasy Mini Handbook from QIAGEN). About 1 x 10^6^ cells were collected, and disrupted in 350 μl of Buffer RLT from QIAGEN (RNeasy Protect Mini Kit) supplemented with 3.5 μl ß-mercaptoethanol. The suspension was homogenized, added to QIAshredder Spin Columns and centrifuged for 2 min at the highest speed. The supernatant was resuspended with 350 μl of ethanol 70% and transferred to RNeasy Mini Spin Columns. A sequence of centrifugations was performed with intermediate washing steps. Total RNA bound to the membrane was incubated with RNase-free DNase and eluted with RNase-free water according to the manufacturer´s instructions. Total RNA was quantified by using a Nanodrop 2000 device (Thermo Scientific, Wilmington, DE). Concentration and purity of samples was verified using Eukaryote Total RNA Nano assay (Agilent Technologies, Santa Clara, CA). To examine RNA integrity and DNA contamination, 1 μg of RNA was electrophoretic separated in an agarose gel. Samples were then saved at -80°C.

#### Microarray hybridization and raw data acquisition

H9c2 cells were collected as previously described and experiments done in triplicate. After RNA extraction and concentration determined, statistical outliers criteria were performed to further analyze sample purification. The microarray GeneChip hybridization was carried at the Genomic Department of the National Center for Cardiovascular Investigation (CNIC) in Madrid, Spain, and further analyzed in the Bioinformatic Unit from the same Institution. Based on the Bioconductor package “ArrayQualityMetrics” there was no evidence of any outlier among the samples. Total rat transcriptome containing about 42,000 transcripts from the Agilent 4x44K chip were identified using Agilent *Rattus norvegicus* genome microarrays. A threshold of 1 and quantiles normalization was applied to the raw signals [13] by using the software GeneSpring. Data were considered in the log2 scale. Default flags were considered as absent, except saturated points that were flagged as marginal.

#### Statistical analyses and gene network analysis

Data analysis was performed by using GraphPad Prism 6.0 program (GraphPad Software, Inc., La Jolla, CA, USA) and data were expressed as means ± SEM for the number of experiments indicated in the legends of the figures. The t-test was used in Western blot method to compare the two groups. A statistically significant difference was considered when p-value < 0.05. Analysis of the enrichment of the biochemical pathways with the differentially expressed transcripts from the RA-treated cells was conducted using The Database for Annotation, Visualization and Integrated Discovery engine (http://david.abcc.ncifcrf.gov). Enrichment p-value lower than 9.9E-3 were taken as significant.

## Results

### Characterization of undifferentiated and RA-differentiated H9c2 cells

**Fig 1:**
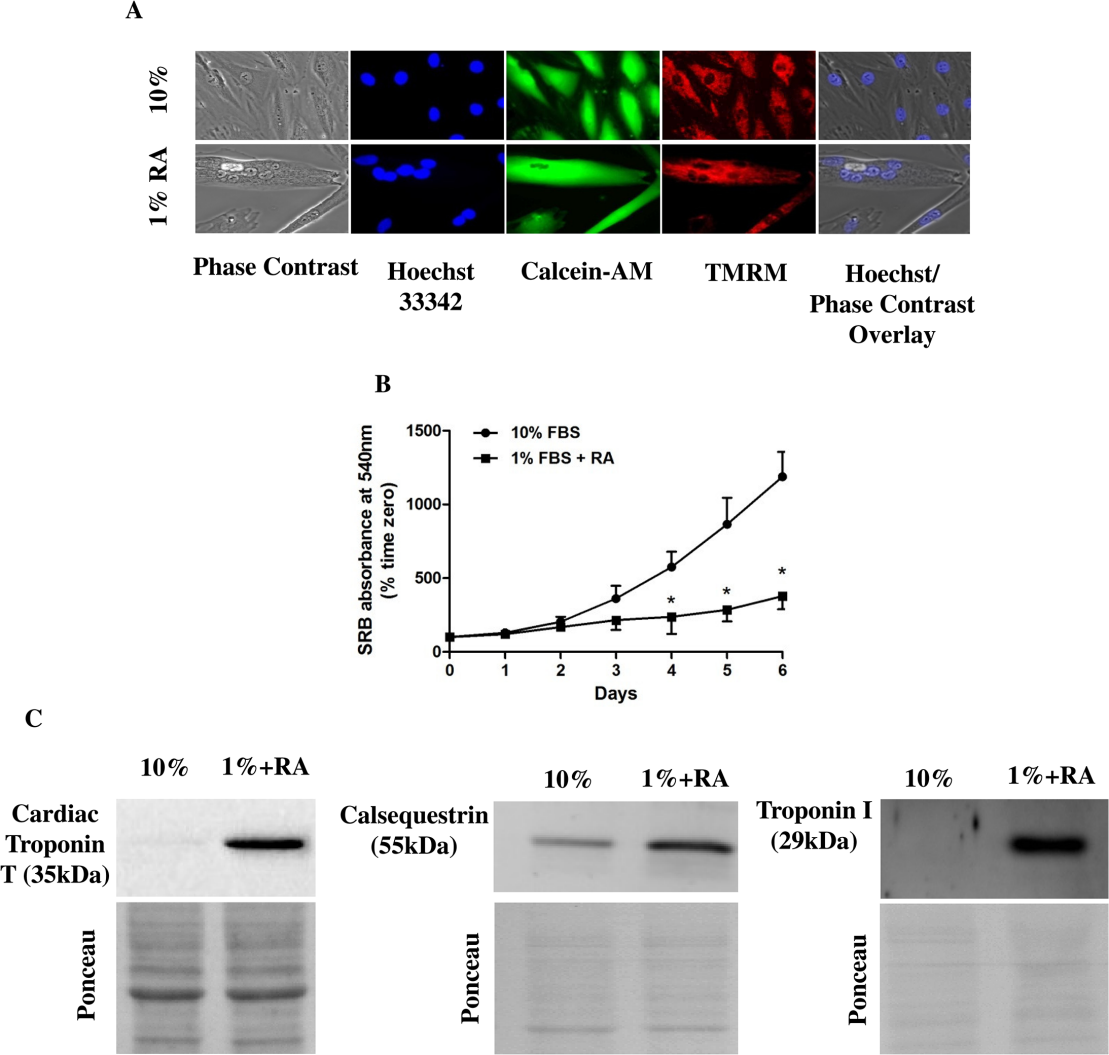
Morphologic and molecular characterization of H9c2 cell line before and after the differentiation process. A) Representative images of the effect of serum withdrawal and RA supplentation on H9c2 cell morphology. Morphologic alterations were observed using the fluorescent probes Hoechst 33342 (nucleus, blue), Calcein-AM (cell viability, green) and TMRM (mitochondria, red). An overlay of phase contrast and Hoechst labeling is also shown. H9c2 cells cultured in 10% FBS-containing medium (top panels) showed a stellar shape, in the most cases, with one nucleus per cell. Upon culture in a medium containing 1% of FBS supplemented with 1 αM RA (bottom panels), differentiated H9c2 cells showed an elongated shape with several nuclei. Epifluorescence microscopy images are representative of 4 different cell preparations. White bar represents 10 μm. B) Decreased proliferative rate by modulating the percentage of serum in the medium. H9c2 cells were seeded at a density of 5,000 cells/ml and allowed to proliferate during 6 days in presence of RA. Results were analyzed by using the SRB assay. Undifferentiated H9c2 cells incubated in a high-serum medium proliferate fast, while incubation in a low-serum medium with daily RA additions during 5 days promoted cell cycle arrest. Statistical analysis: * p < 0.05 vs 10% FBS; the graph is representative of 5 independent experimental studies. C) Cellular content of specific cardiac marker troponin T, troponin I and cardiac calsequestrin in different cell populations. By using Western blotting, it was possible to confirm that RA-differentiated cells have increased content of cardiac markers. The images are representative of 4 different experimental studies which produced the same result. Ponceau labeling represents the loading control and was used to normalize data.

With the objective of characterizing H9c2 cell cardiac-driven differentiation, a simple visual assessment by microscopy was performed in H9c2 myoblasts growing on medium supplemented with 10% FBS (growth-promoting medium) or in differentiated H9c2 cells maintained in medium supplemented with 1% of FBS plus 1 μM RA (Fig 1).

When cultured in high-serum medium, H9c2 cells are commonly mononucleated and exhibit spindle-to-stellate shape (Fig 1A, top panels). However, incubation of H9c2 cells during 5 days in low serum medium in the presence of RA, induced cell fusion and the formation of larger multinucleated cells (Fig 1A, bottom panels), a morphological indication of H9c2 differentiation. In order to verify whether H9c2 differentiation promotes alterations in cellular viability and mitochondrial morphology, cells were incubated with the mitochondrial-selective probe TMRM and with fluorescent probes Hoechst 33342 and Calcein-AM. As shown in Fig 1A (right panels), both undifferentiated and differentiated myoblasts present a large filamentous mitochondrial network. Calcein-AM labeling shows that the vast majority of cells in both groups are viable thus retaining the dye.

To assess the effect of serum withdrawal on H9c2 cells proliferation, we measured cell mass during 6 days in culture by using the colorimetric SRB method. The data confirm a reduction in the cell proliferation rate when H9c2 cells were cultured in low serum medium supplemented with RA, with more evident differences during the growth exponential phase (Fig 1B).

We next investigated whether H9c2 differentiation was accompanied by increased expression of cardiac-specific markers. Higher content in cardiac troponin T, troponin I and cardiac calsequestrin were observed when H9c2 cells were differentiated in a low serum media supplemented with RA (Fig 1C), confirming a cardiac-like phenotype, as also shown previously by our group [6, 9].

### Gene expression profiling alterations during cardiac-driven differentiation of H9c2 myoblasts

To obtain an alternative snapshot of H9c2 cell remodeling during the differentiation process, we performed a comparative gene expression analysis by using microarray technology to identify differences in gene transcription. Oligonucleotide microarray and bioinformatics technology are widely used in identifying transcription factors that regulate cellular processes such as cell differentiation. In this study, a high coverage of the transcriptome data was performed with the objective of sorting out differentially expressed transcripts during H9c2 differentiation. The more significant hits were further investigated regarding protein-encoding levels and related signaling pathways to better characterize regulation mechanisms of differentiation in this cell line. After correction of the data and identification of significant differences on gene regulation (based on the adjust p-value < 0.05), we identified several transcriptional alterations. We identified 3,433 gene transcripts that were differently expressed between non-differentiated and differentiated H9c2 cells. The remaining genes showed mild changes on expression level (<2-fold) and were not further considered in the study. Complex analysis of the biochemical and genetic pathways enriched with genes whose expression was altered in RA-differentiated cells confirmed activation of cardiac-specific pathways. Instead, pathways related to cell cycle, DNA repair and cell maturation were suppressed, confirming the progress to cell differentiation (Table 1).

**Table 1:** Metabolic and signaling pathways represented by the alternatively expressed gene transcripts in RA-differentiated cells.

Activated cascades			Suppressed cascades		
Pathway	Number of genes	P value	Pathway	Number of genes	P value
Steroid biosynthesis	10	7.3E-8	DNA replication	22	7.6E-20
Terpenoid backbone biosynthesis	8	3.7E-6	Cell cycle	37	1.4E-19
Dilated cardiomyopathy	18	6.6E-6	Mismatch repair	12	9.1E-10
Hypertrophic cardiomyopathy (HCM)	17	1.1E-5	Homologous recombination	11	1.2E-7
Cardiac muscle contraction	14	3.1E-4	Nucleotide excision repair	12	3.0E-6
Arrhythmogenic right ventricular cardiomyopathy (ARVC)	12	2.3E-3	Oocyte meiosis	18	1.4E-5
p53 signaling pathway	11	3.1E-3	Pyrimidine metabolism	15	8.5E-5
Glutathione metabolism	9	5.7E-3	Base excision repair	9	3.1E-4
Steroid hormone biosynthesis	8	8.7E-3	Progesterone-mediated oocyte maturation	13	6.9E-4
Biosynthesis of unsaturated fatty acids	6	9.0E-3	Purine metabolism	18	8.3E-4

The pool of significantly up- and down-regulated genes in RA-differentiated cells was analyzed for the specific enrichment in any biochemical pathways using DAVID Bioinformatics Resources engine and KEGG-based database. Top 10 pathways showed significant enrichment with up-regulated and down-regulated genes in the differentiated cells are shown.

Thus, results from microarray gene expression analyses confirm that differentiation of H9c2 cells towards a cardiac phenotype led to an expected increase in cardiac-specific mRNAs. Several transcripts identified in this study are intrinsically associated with differentiation processes, proliferative capacity, signal transduction and energy processing mechanisms, supporting that mitochondrial energy production are increasing its importance as cardiac cells differentiate into an adult phenotype [9, 14]. Although the analysis revealed several sets of genes differentially expressed between undifferentiated and RA-differentiated samples, only a few genes were further analyzed (Table 2).

**Table 2:** Most relevant differences in terms of gene expression in H9c2 undifferentiated myoblasts vs RA-differentiated H9c2 cells.

Differentiated cells vs Non-differentiated cells			
Function	Gene	Name	FC
Dif	Tnnt2	troponin T type 2 (cardiac)	4.48
Dif	Myog	myogenin	9.45
Dif	Myom2	myomesin 2	32.62
CellSig	Bcl2	B-cell CLL/lymphoma 2	0.35
CellSig	Faslg	Fas ligand (TNF superfamily, member 6)	17
CellSig	Bcl2l11	BCL2-like 11 (apoptosis facilitator)	2.07
Metabol	Ucp2	uncoupling protein 2 (mitochondrial, proton carrier)	2.05
Metabol	Ucp3	uncoupling protein 3 (mitochondrial, proton carrier)	10.96
Metabol	Rbp2	retinol binding protein 2	5.2
Metabol	Cpt1a	carnitine palmitoyltransferase 1a	2.13
Metabol	Ckmt1	creatine kinase, mitochondrial 1	2.99
Metabol	Ckmt2	creatine kinase, mitochondrial 2, sarcomeric	7.11
Metabol	Pdk2	pyruvate dehydrogenase kinase, isozyme 2	2.12
Metabol	Ppargc1b	peroxisome proliferator-activated receptor gamma	2.69
Metabol	Ldhb	lactate dehydrogenase B	5.57
Metabol	Cox6a2	cytochrome c oxidase, subunit VIa, polypeptide 2	6.40
Metabol	Cox8b	cytochrome c oxidase, subunit VIIIb (Cox8b)	9.48
Metabol	Ndufs4	NADH dehydrogenase (ubiquinone) Fe-S protein 4	2.1
Metabol	Gpx1	glutathione peroxidase 1	0.2
Metabol	Slc16	monocarboxylate transporter 1 (MCT 1)	2.12
Cellcycle	Rb1	retinoblastoma-like 1 (p107)	0.1
Cellcycle	E2f1	E2F transcription factor 1	0.26
Cellcycle	Hdac1	histone deacetylase 1	0.49
Cellcycle	Bard1	BRCA1 associated RING domain 1	0.11
Cellcycle	phb	prohibitin-2	0.47
Cellcycle	Mybl2	myeloblastosis oncogene-like 2	0.09
Calcium	Pde4a	phosphodiesterase 4A, cAMP-specific	0.44
Calcium	Atp2a2	ATPase, Ca transporting, cardiac muscle, slow twitch	2.75
Calcium	Atp2a1	ATPase, Ca transporting, cardiac muscle, fast twitch 1	14.42
Calcium	Pln	phospholamban	3.63
Calcium	Sln	sarcolipin	23.21
Calcium	Ryr1l	ryanodine receptor type 1	12.8

**Fig 2:**
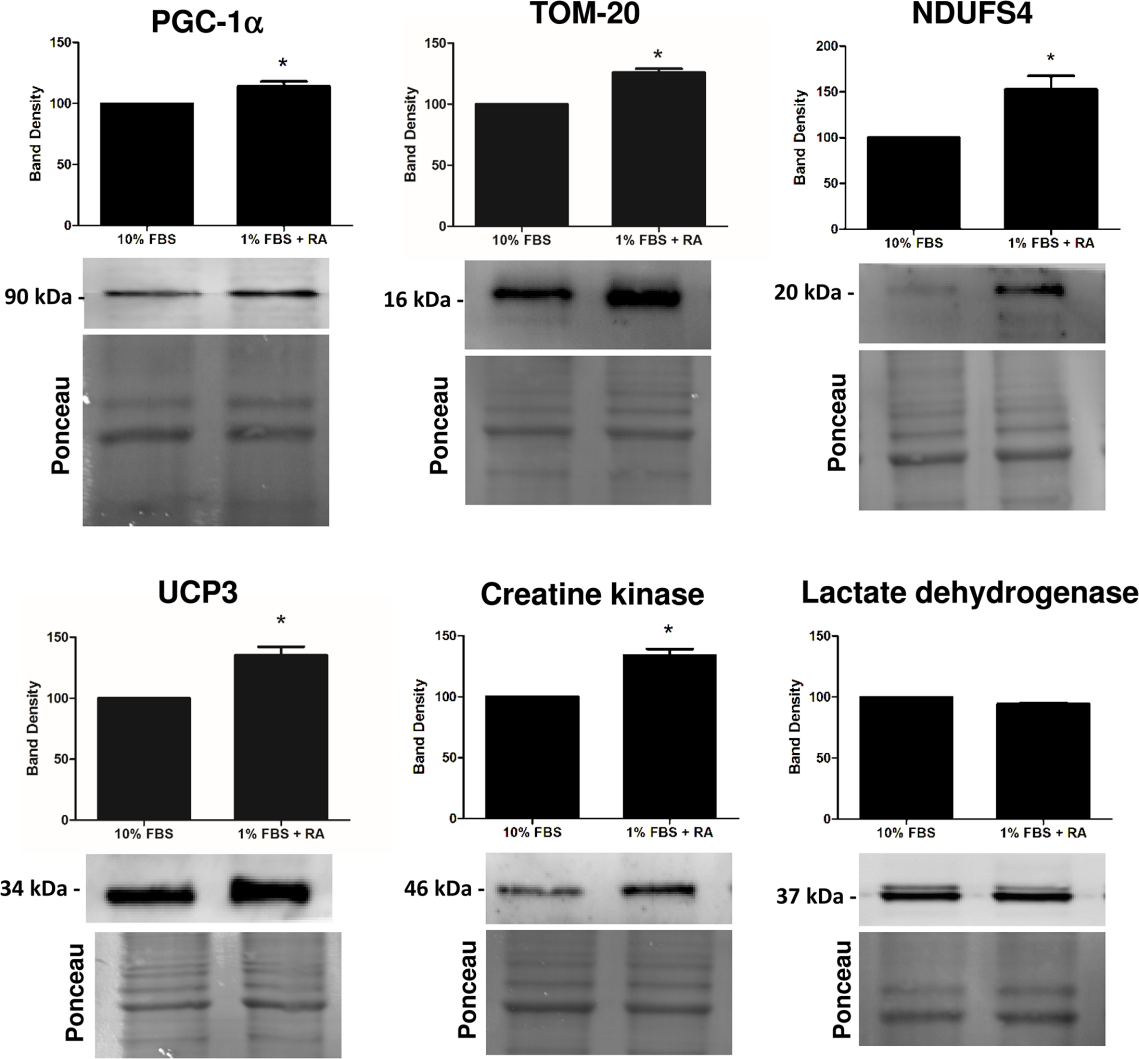
Semi-quantification by Western blotting of selected proteins involved on mitochondrial function and metabolism. Protein content of PGC1α, TOM20, complex I NDUFS4 subunit, uncoupling protein 3, creatine kinase and lactate dehydrogenase was measured as described in the materials and methods section. H9c2 myoblasts were differentiated by being cultured in 1% FBS medium supplemented with 1 μM RA and maintained for 5 days. Total extracts from both cellular groups were collected. Ponceau labeling represents the loading control and was used to normalize data. Data represent the mean ± SEM of 4–5 independent experiments (*) p<0.05 versus respective undifferentiated group.

H9c2 cells were differentiated for 5 days in a low serum concentrated medium daily supplemented with RA. Both groups, undifferentiated and RA-differentiated cells, were collected and analyzed as described in Material and Methods section. FC>2 corresponds to, at least, two-fold up-regulation of the gene, FC<2 corresponds to, at least, two-fold down-regulation of the gene in differentiated cells with RA. Data correspond to four separate experiments and differences are also represented in Fig 2. Abbreviation: Dif–genes related with differentiation process to a cardiac phenotype; CellSig–genes encoding proteins related with cell signaling pathways; Metabol—genes related with metabolic activity; Cellcycle—genes related with proliferation activity; Calcium–genes related with cellular calcium handling.

RA-differentiated H9c2 cells showed increased expression of genes related with calcium transporters (such as the sarcoplasmic reticulum calcium ATPase–SERCA, phospholamban sarcolipin and ryanodine receptor), cardiac sarcomeric proteins such as troponin T and troponin C and differentiation markers such as myogenin, a transcription factor essential for terminal differentiation into myocytes [15]. Increased transcripts for retinol binding protein 2 (Rbp2) were found in differentiated cells, which may impact the availability/signaling effects of RA that result downstream in cell differentiation.

Regarding cell energy production, RA-differentiated H9c2 cells showed increased expression of genes relevant for mitochondrial energy production including mitochondrial creatine kinase, cytochrome c oxidase subunits COX6a2, COX8b and COX11, uncoupling proteins 2 and 3, Complex I subunit NADH dehydrogenase (ubiquinone) Fe-S protein 4 (NDUFS4), carnitine palmitoyltransferase 1a, pyruvate dehydrogenase kinase 2, lactate dehydrogenase B, monocarboxylate transporter 1 and the peroxisome proliferator-activated receptor gamma coactivator-1 beta which is involved in cardiac mitochondrial biogenesis. Transcripts of genes involved in cellular death signaling pathways such as FasL and Bcl2L11, involved in the inhibition of cell cycle progress and apoptosis, were also augmented in RA-differentiated H9c2 cells. On the other hand, undifferentiated myoblasts show increased transcripts for pro-survival proteins such as Bcl-2 and the antioxidant protein glutathione peroxidase 1. Also, genes playing important roles in the regulation of cell cycle including cyclin A were also up-regulated in undifferentiated myoblasts. Undifferentiated myoblasts showed increased transcripts for the E2F transcription factor 1, HDAC1, BRCA1-associated RING domain 1, prohibitin-2 and mybl2. Moreover, transcripts for the cAMP-specific phosphodiesterase 4A and mitogen-activated protein kinase, were also up-regulated in undifferentiated cells.

The results indicate that the differentiation of H9c2 cells obtained with serum decrease and RA addition led to a transcriptional up-regulation of genes involved in cardiac differentiation.

### Protein content evaluation after the differentiation process

**Fig 3:**
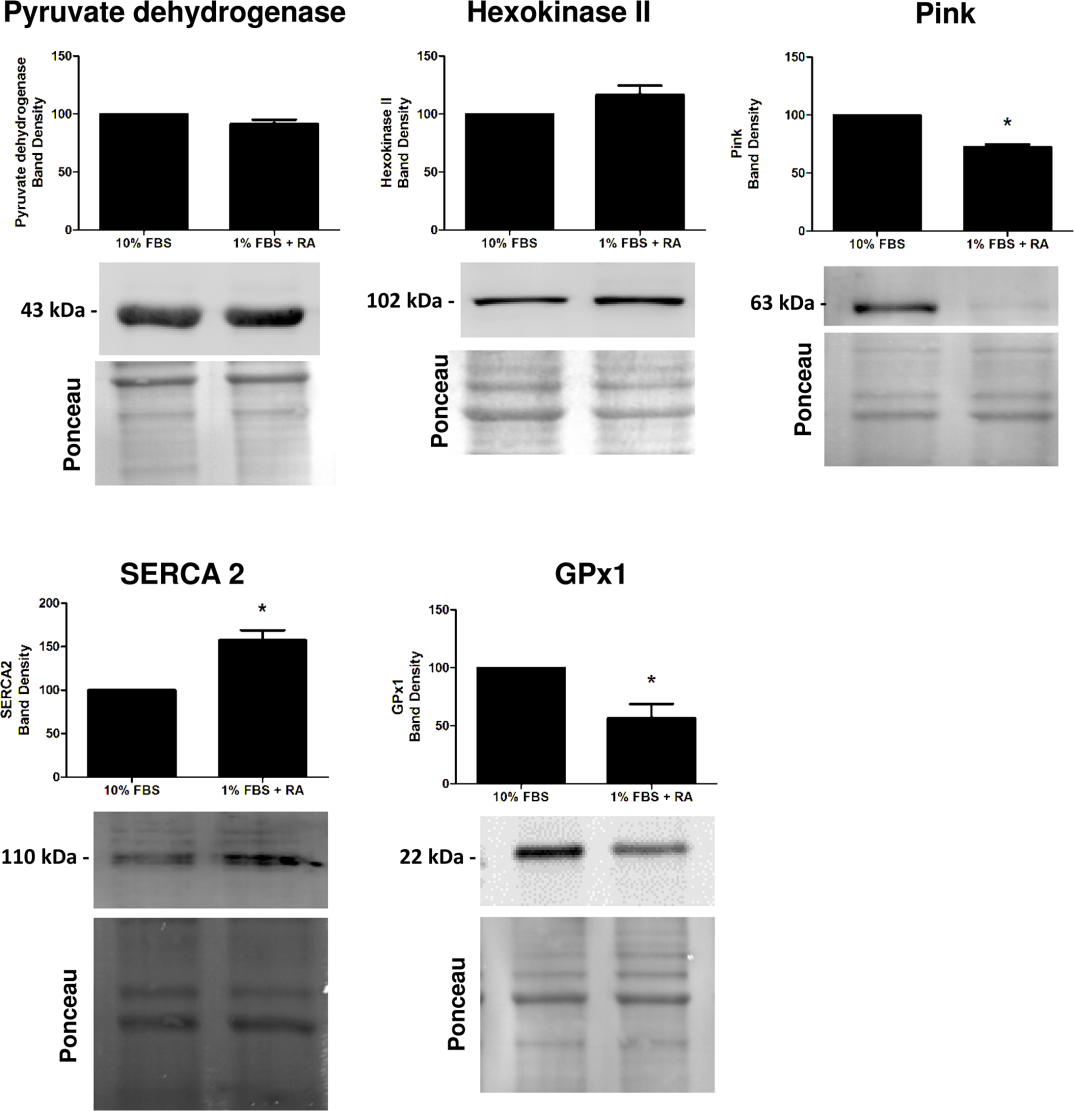
Semi-quantification of selected proteins involved on calcium handling and metabolism by Western blotting. Protein content of pyruvate dehydrogenase, hexokinase II, Pink, SERCA2 and glutathione peroxidase 1 was measured by Western blotting as described in the materials and methods section. H9c2 myoblasts were differentiated by being cultured in 1% FBS medium supplemented with 1 μM RA and maintained for 5 days. Total extracts from both cellular groups were collected. Ponceau labeling represents the loading control and was used to normalize data. Data represent the mean ± SEM of 4 independent experiments (*) p<0.05 versus undifferentiated group.

**Fig 4:**
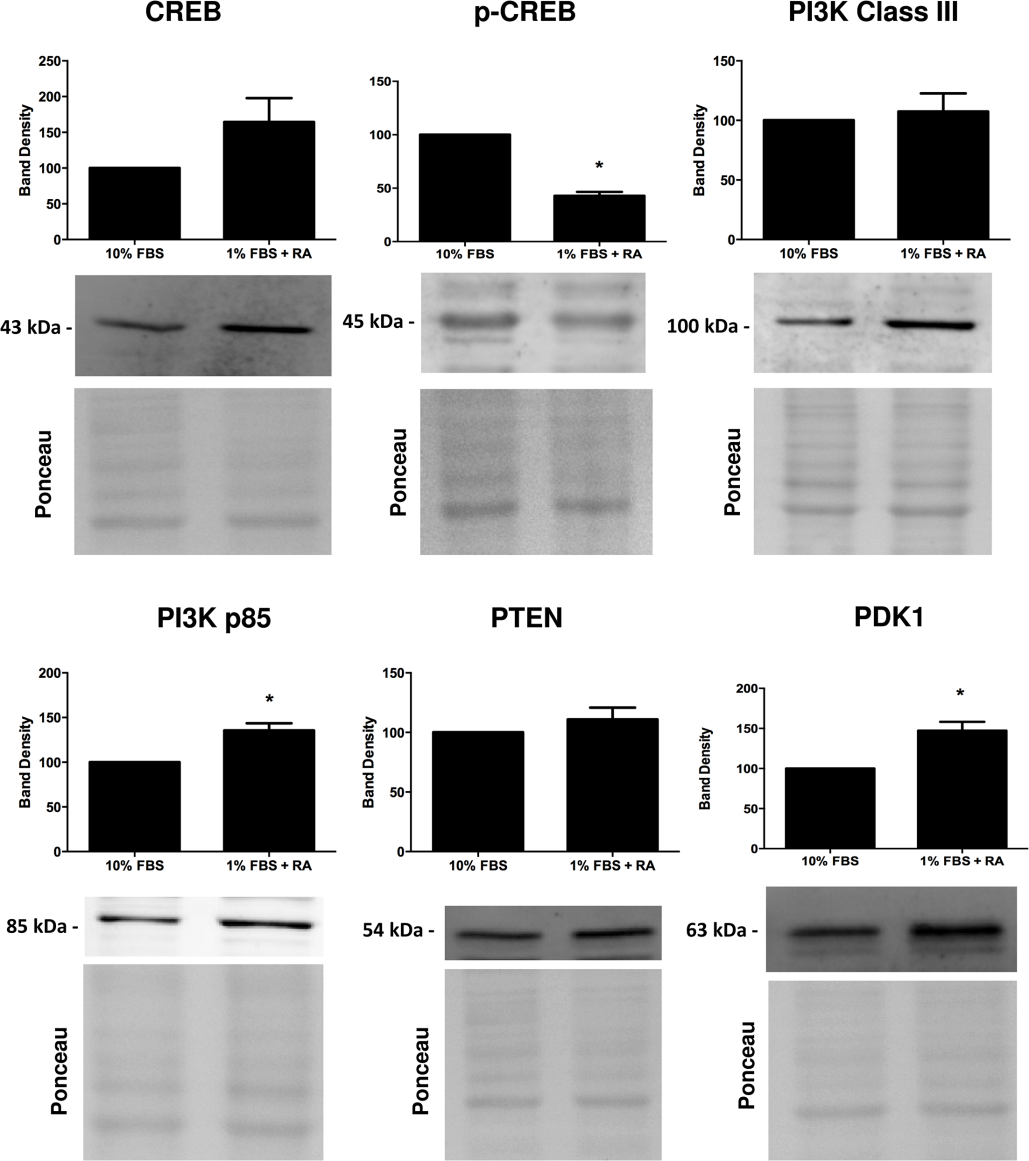
Evaluation by Western blotting of selected proteins with probable involvement on the differentiation of H9c2 cells towards a cardiac-like cells. Cellular content of Creb (total and phosphorylated form), PI3K (Class III and p85), PTEN and PDK1were measured as described in the materials and methods section. Ponceau labeling represents the loading control and was used to normalize data. Data represent the mean ± SEM of 4 independent experiments (*) p<0.05 vs undifferentiated group.

**Fig 5:**
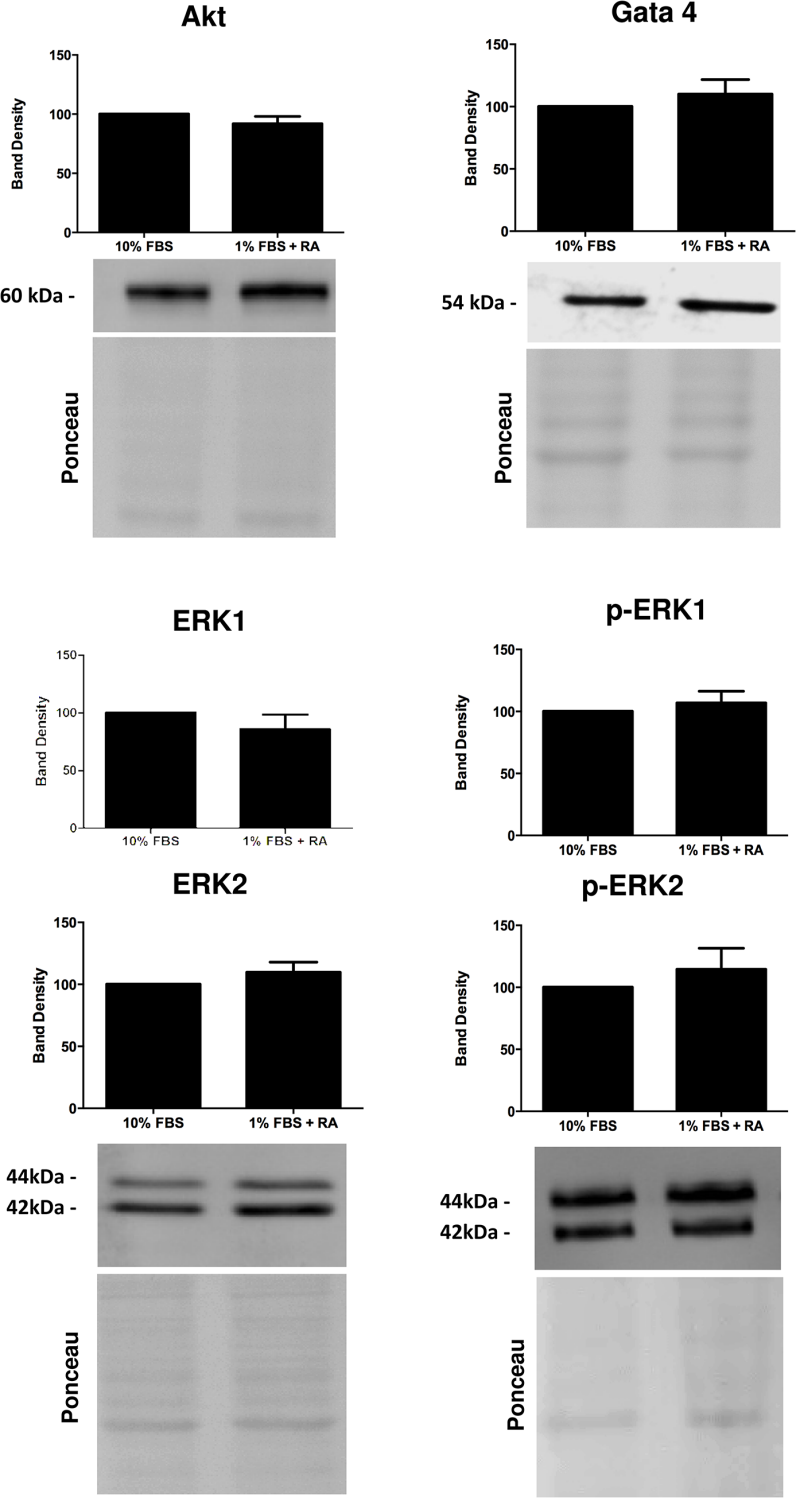
Evaluation by Western blotting of selected proteins with probable involvement on the differentiation of H9c2 cells towards a cardiac-like cells. Cellular content of Akt, Gata 4, ERK1 (total and phosphorylated), and ERK2 (total and phosphorylated) were measured as described in the materials and methods section. Ponceau labeling represents the loading control and was used to normalize data. Data represent the mean ± SEM of 4 independent experiments (*) p<0.05 vs respective undifferentiated group.

The evaluation of results from microarray analysis gives helpful information in identifying specific pathways involved in the cardiac differentiation process. In this regard, alterations in proteins resulting from the differential transcriptome profile are critical to understand the biochemical remodeling during the cell differentiation process. Taking this into context, we next used Western blotting to semi-quantify some of the relevant proteins related with cell metabolism and calcium handling (Figs 2 and 3), as well with signaling pathways that may be activated during the differentiation process (Figs 4 and 5).

We observed that H9c2 cardiomyoblast differentiation is accompanied by an increase in the amount of PGC1α, TOM20, NDUFS4, UCP3, creatine kinase, SERCA2, PI3K, p85 and PDK1 and with a decrease in the antioxidant protein glutathione peroxidase 1 (GPx1), Pink1 and in phospho-CREB. No differences dependent on the cell differentiation state were observed regarding total lactate dehydrogenase, hexokinase II, pyruvate dehydrogenase (PDH), GATA 4, total ERK (ERK 1/2), phosphor-ERK, PI3K class III, CREB, PTEN and Akt proteins.

## Discussion

The rat H9c2 cell line is an established *in vitro* model for cardiomyocytes in a wide variety of experimental studies [16, 17]. H9c2 cells are a relevant model to investigate mechanisms and consequences of different cardiac pathologies, including cardiac hypertrophy [5]. Despite the ability of H9c2 myoblasts to differentiate in adult muscle cells, the majority of toxicological studies involving this cell line is performed with undifferentiated myoblasts. In this regard, it is necessary to clarify whether the use of undifferentiated H9c2 cells is the best model for adult cardiomyocytes. Since H9c2 myoblasts have the capacity to differentiate into skeletal and cardiac muscle cells, their use on developmental toxicology studies increased over the years. Several reports documented that the differentiation state of the cardiac cell impacts its susceptibility to a toxicant [6, 7, 18]. In this regard, it is of great interest to understand the molecular basis of cellular changes that occur during H9c2 cardiomyoblast differentiation.

Specific alterations commonly accompany muscle cell differentiation in vivo, including cell cycle arrest and morphological alterations such as the formation of multinucleated myotubes. The results obtained in this study confirm that H9c2 myoblasts fuse and form multinucleated cells after decreasing the media serum to 1% in the presence of RA supplementation (Fig 1A). It has been previously shown that the differentiation process initiates a cell cycle arrest, which decreases cellular proliferation, deriving from removal of medium growth factors [19]. The addition of RA drives the differentiation towards a cardiac phenotype. In fact, although the differentiation protocols yield heterogeneous populations [4], Fig 1C shows that RA-differentiated cells present a clear higher content in troponin T, troponin I and calsequestrin, markers for cardiac differentiation [20].

Gene arrays of the entire cell genome showed that expression of multiple markers of cardiomyogenesis occurs during RA-drive differentiation, confirming Western blotting data (Fig 1). Complex analysis of the pool of genes significantly up- and down-regulated in the RA-treated cells showed clear enrichment in activated genetic pathways associated with cardiac phenotype. Instead, pathways associated with cell cycle, DNA repair and cell division were enriched with down-regulated genes (Table 1). Taking together, these results strongly suggest the successful RA-driven cardiomyoblast differentiation towards a more adult muscle phenotype.

Specifically, increased Myog and Myom2 transcript levels after RA-differentiation also demonstrate a clear transition to a more mature and adult muscle cell. As mentioned previously, modulation of signaling proteins during cell differentiation cause a higher susceptibility of a specific developmental stage of the tissue to a cardiotoxic agent. Of particular importance, we found alterations in transcripts (and later in protein) that may impact cellular responses to a toxicant. Alterations in cell cycle, calcium-modulating or metabolic proteins during the differentiation process can alter the susceptibility of H9c2 cells to toxicants. As expected, transcripts with important roles in the regulation of cell cycle were more present in undifferentiated cells. Retinoblastoma like 1-encoded or related genes, such as the E2F transcription factor 1, HDAC1, BRCA1 associated RING domain 1, prohibitin-2 and mybl2 were up-regulated in undifferentiated myoblasts.

Gene expression for pro- and anti-apoptotic proteins were also identified. The gene array analysis confirmed increased transcripts for Bcl-2, a pro-survival protein, on undifferentiated cells. Moreover, genes involved in death signaling pathways such as the FasL and the Bcl2L11, which favor apoptosis, were also increased in RA-differentiated H9c2 cells (Table 2). The balance between pro and anti-apoptotic proteins is crucial for the cellular response to a stressor agent. It has been demonstrated by our group that RA-differentiated cells present higher susceptibility to various cardiotoxicants which can be, at least in part, due to more active survival mechanisms in the undifferentiated cellular pool [6, 7]. The decrease in Pink1 protein found in differentiated cells (Fig 3) may also explain differences regarding the susceptibility of those cells to cell death mediated by mitochondria. Pink1 is a mitochondrial outer membrane protein, highly expressed in the heart, which protects cells from stress-induced mitochondrial dysfunction. Pink1 is involved in mitophagic processes that remove and replace damaged mitochondria. Since differentiated H9c2 cells have increased oxidative machinery and metabolism [9], a decrease in a mitophagy-related protein may imply that damage to those structures may not always be corrected.

The present transcriptional analysis reveals that RA-differentiated myoblasts have increased expression of genes related with mitochondrial function, including respiratory chain complexes and uncoupling proteins, confirming our previous results that RA-driven H9c2 cell differentiation towards a more cardiac-like phenotype involves remodeling and up-regulation of mitochondrial function [9]. More transcripts for uncoupling protein 2 and 3 (as well as protein in this letter case) in RA-differentiated H9c2 cells may be a compensatory response to an increase in mitochondrial activity after cell differentiation. Uncoupling proteins have important roles in fatty acid β-oxidation and mitochondrial oxidative stress [21]. In fact, when fatty acid delivery to mitochondria overwhelm the oxidative capacity, the expression of UCP3 increases leading to reduced storage of non-esterified fatty acid [22]. Non-esterified fatty acid anions have deleterious effects on mitochondria because they are non-metabolized and, in this regard, UCP3 exerts a protective role in their export from mitochondria [21]. Interestingly, the Cpt1 gene that encodes for the carnitine palmitoyltransferase 1 was also increased in RA-differentiated cells (Table 1). This shuttle system is responsible for the transport of fatty acyl-CoA esters from the cytosol to mitochondria for β-oxidation metabolism [21]. This enzyme is controlled by acetyl-coenzyme A carboxylase, playing a role in mitochondrial fatty acid uptake and oxidation as well as in creatine kinase regulation [23]. Still concerning energy production, mitochondrial creatine kinase was more expressed in differentiated cells (Table 1, Fig 2). This enzyme plays a pivotal role in muscle function due to its capacity of energy storage, explaining its higher presence in tissues with high energy demand [24]. Higher transcript content in differentiated cells was further confirmed by semi-quantifying protein levels by Western blotting. In accordance with our findings regarding gene expression, it was previously demonstrated that AMP deaminase as well as creatine kinase activities were more than 10-fold elevated in differentiated H9c2 cells [25].

Cardiogenesis is associated with significant alterations in the expression of pyruvate dehydrogenase kinase (PDK), a pyruvate dehydrogenase (PDH) regulator [26]. PDH is a regulatory site in cellular metabolism linking the TCA cycle and oxidative phosphorylation with glycolysis, facilitating pyruvate oxidation and substrate supply to distinct parts of the mitochondrial network. In the present study, increased PDK2 transcripts were found in RA-differentiated cells, which lean toward a more oxidative metabolism [9]. PDK2 suppresses PDH activity, which converts pyruvate into acetyl-CoA, a fundamental product used by TCA cycle enzymes. As PDH activity commonly follows mitochondrial respiration rates [26], which increase during cardiac differentiation, a higher PDH activity, hence low PDK2 activity, would be expected in more differentiated cells. Not only were the activity of PDK2 and PDH not measured in this work, as also PDK vary greatly in their kinetic and post-translational properties [27, 28]. Interestingly, RA-differentiated cells also showed increased transcripts for acetyl-CoA-related enzymes, including acetoacetyl-CoA synthetase, acetyl-CoA carboxylase or acetyl-CoA acetyltransferase 2 (Table 1).

Lactate dehydrogenase B transcripts increased in differentiated cells, in concert with the observed transcript expression of MCT1, a monocarboxylase transporter (Table 1), suggesting alterations in lactate/pyruvate metabolism, although no significant alterations were observed when both subunits of lactate dehydrogenase were analyzed by Western blotting.

The results also confirmed up-regulation of mitochondrial genes in RA-differentiated cells, including cytochrome c oxidase subunits COX6a2, COX8b and COX11 and complex I subunit NADH dehydrogenase (ubiquinone) Fe-S protein 4, with some of those later confirmed by Western blotting analyses. Interestingly, the gene coding for the translocase of inner mitochondrial membrane (TIM) was found increased in undifferentiated cells; however, protein levels of the translocase of outer mitochondrial membrane (TOM) was increased in RA-differentiated cells (Table 1, Fig 2). The PGC-1 family (PGC-1α, PGC-1β, and PRC) plays a critical role in mitochondrial biogenesis as overexpression of these proteins results in increased mitochondrial content and in a more oxidative muscle subtype [29]. PGC-1β transcript and protein was increased in RA-differentiated samples, suggesting increased mitochondrial biogenesis. Furthermore, the overexpression of PGC-1β induces its conversion to highly oxidative “fast-twitch” muscle fibers rich in mitochondria [30]. In what concerns to antioxidant defenses, GPx1 transcript and protein is increased in undifferentiated cells suggesting a higher degree of protection from oxidative damage on this cell group. Although contributing to an up-regulated mitochondrial metabolism, the increase in several mitochondrial-related proteins and activity (Fig 3 and [8]) in RA-differentiated cells, may also contribute to increased generation of reactive oxygen species (ROS). The observed decrease in GPx1 as well as in Pink1 proteins (Fig 3) in RA-differentiated cells may suggest a less efficient antioxidant and quality control mechanism.

There is evidence that phosphodiesterases (PDE) activity decreases with age [31]. Here, we found that PDE is more expressed in undifferentiated cells. In the rodent heart, PDE4 contributes up to 60% of total cAMP hydrolyzed [32]. As described by others, inhibition of PDE increases cAMP and consequently increase intracellular calcium levels through activation of calcium channels [33]. This is in accordance with our results in which an increase in basal intracellular calcium levels in RA-differentiated cells was found [8]. Genes encoding for calcium transporters and regulators such as the sarcoplasmic reticulum calcium ATPase-SERCA1 and 2, its regulator phospholamban (PLB), sarcolipin, and ryanodine receptors also experienced alterations during cellular differentiation. An increase in SERCA2 transcript and protein was found in differentiated cells, suggesting more active calcium regulating mechanisms. Regarding this, calcium/calmodulin-dependent kinase plays a central role in the susceptibility of differentiated H9c2 cells after exposure to isoproterenol [8].

Several signaling pathways are presumably involved in the differentiation process of H9c2 myoblasts towards a more cardiac phenotype. Previous studies have indicated a role of RA-regulating mitogen-activated proteins kinases pathways [34]. Our results (Figs 4 and 5) suggest MAP kinase as likely involved in the differentiation process. We previously observed a significant increase in p38 MAK Kinase, complemented by our current observation that a tendency for increased ERK and phospho-ERK exist during H9c2 cell differentiation. ERK 1/2, p38 and JNK1/2 can be regulated by MKP-1, a member of the dual-specificity phosphatase family that is expressed in the heart, where it regulates de activity of these proteins. To explore the molecular mechanisms by which RA induces the differentiation of H9c2 in low serum medium into a cardiac-cell line, we investigated by Western blotting selected MAPK family proteins which have a role in cardiac differentiation, namely PI3K, CREB and Mitogen-activated protein kinases (MAPK) family-related. We observed a significant increase in PI3K p85 and PDK1 and a decrease in the phosphorylated form of CREB on serine 133 upon H9c2 differentiation.

Gata-4, a transcription factor expressed in cardiac cells and activated by p38-MAPK [35] showed no changes after differentiating H9c2 cells with RA (Fig 5). Similar results were found for total and phosphorylated forms of ERK 1 and 2. PI3K was expected to be a good candidate as it was documented that p38-MAPK can be downstream to PI3K activation [36]. We found an increase in the regulatory domain of PI3K and no changes on the phosphatase and tensin homolog deleted on chromosome 10 protein (PTEN), a phosphatase that convert PIP3 to PIP2 (the reverse reaction regulated by PI3K). Interestingly, levels of PDK1, protein activated by PIP3, were found increased in RA-differentiated cells. This kinase phosphorylates Akt leading to its activation, which in turn regulates other proteins and transcriptional factors including CREB [37]. Since our results showed a decrease in the p-CREB after RA-differentiation, and although PI3K and PDK1 protein is increased, it is likely that CREB activity is decreased, leading cells to stop proliferating.

The major objective of this work was to perform a transcriptional analysis of H9c2 differentiation, in order to legitimate the potential use of H9c2 cells as a model for cardiac cells in different studies, including toxicology. We have identified a large number of genes with altered expression during differentiation towards a more cardiac phenotype, pointing out to an augmented oxidative metabolism and calcium handling. To our knowledge, this is the first report of alterations of the H9c2 cell transcriptome during differentiation, which impacts our understanding of how this cell line can be used as an adequate cardiac model.

The data here obtained suggests that differentiated H9c2 cells can be a better model for cultured primary cardiomyocytes than non-differentiated cells. Facing its low cost of maintaining in culture, plus avoiding the need to sacrifice animals, combined with the existence of cardiac-specific markers and protein/metabolic signatures that resemble adult cardiac cells, makes this a suitable cell line to potentially substitute cultures cardiomyocytes in toxicology studies.

Conflicts of interest

The authors report no conflicts of interest.
